# Effect of the consumption of a fermented dairy product containing *Bifidobacterium lactis *DN-173 010 on constipation in childhood: a multicentre randomised controlled trial (NTRTC: 1571)

**DOI:** 10.1186/1471-2431-9-22

**Published:** 2009-03-18

**Authors:** Merit M Tabbers, Ania Chmielewska, Maaike G Roseboom, Claire Boudet, Catherine Perrin, Hania Szajewska, Marc A Benninga

**Affiliations:** 1 Department of Paediatric Gastroenterology and Nutrition, Emma's Children's Hospital/Academic Medical Centre, Amsterdam, The Netherlands; 2 2nd Department of Paediatrics, The Medical University of Warsaw, Warsaw, Poland; 3 Altran, Levallois-Perret, France; 4 Altran, Danone Research, Palaiseau, France

## Abstract

**Background:**

Constipation is a frustrating symptom affecting 3% of children worldwide. Randomised controlled trials show that both polyethylene glycol and lactulose are effective in increasing defecation frequency in children with constipation. However, in 30–50%, these children reported abdominal pain, bloating, flatulence, diarrhoea, nausea and bad taste of the medication. Two recent studies have shown that the fermented dairy product containing *Bifidobacterium lactis *strain DN-173 010 is effective in increasing stool frequency in constipation-predominant irritable bowel syndrome patients with a defecation frequency < 3/week and in constipated women with a defecation frequency < 3/week. Goal of this study is to determine whether this fermented dairy product is effective in the treatment of constipated children with a defecation frequency < 3/week.

**Methods/design:**

It is a two nation (The Netherlands and Poland) double-blind, placebo-controlled randomised multicentre trial in which 160 constipated children (age 3–16 years) with a defecation frequency <3/week will be randomly allocated to consume a fermented dairy product containing *Bifidobacterium lactis *DN-173 010 or a control product, twice a day, for 3 weeks. During the study all children are instructed to try to defecate on the toilet for 5–10 minutes after each meal (3 times a day) and daily complete a standardized bowel diary. Primary endpoint is stool frequency. Secondary endpoints are stool consistency, faecal incontinence frequency, pain during defecation, digestive symptoms (abdominal pain, flatulence), adverse effects (nausea, diarrhoea, bad taste) and intake of rescue medication (Bisacodyl). Rate of success and rate of responders are also evaluated, with success defined as ≥ 3 bowel movements per week and ≤1 faecal incontinence episode over the last 2 weeks of product consumption and responder defined as a subject reporting a stool frequency ≥ 3 on the last week of product consumption. To demonstrate that the success percentage in the intervention group will be 35% and the success percentage in the control group (acidified milk without ferments, toilet training, bowel diary) will be 15%, with alpha 0.05 and power 80%, a total sample size of 160 patients was calculated.

**Conclusion:**

This study is aimed to show that the fermented dairy product containing *Bifidobacterium lactis *strain DN-173 010 is effective in increasing stool frequency after 3 weeks of product consumption in children with functional constipation and a defecation frequency < 3/week.

## Background

Chronic constipation is a common problem in childhood with an estimated prevalence of 3% in the western world [1]. Constipation is a debilitating condition characterized by infrequent painful defecation, faecal incontinence and abdominal pain. It causes distress to child and family and results in severe emotional disturbance and family discord. The pathophysiology underlying functional constipation is undoubtedly multi-factorial, and not well understood. Withholding behaviour is probably the major cause for the development of constipation and might be caused by the previous production of a large, hard painful stool, anal fissures, a primarily behavioural mechanism or the resistance to go to another toilet than their own [2]. A study in a tertiary hospital showed that despite intensive medical and behavioural therapy, 30% of the patients who developed constipation before the age of 5 years continued to have severe complaints of constipation, infrequent painful defecation and faecal incontinence, beyond puberty [3].

Presently, there is no proper therapy available, mainly due to a lack of pathophysiological insight. In a recently published systematic review on the effect of laxative treatment and dietary measures in childhood constipation, the authors found insufficient evidence supporting that laxative treatment is better than placebo in children with constipation due to a lack of placebo controlled trials [4]. Furthermore, RCT's performed in children with constipation showed that both polyethylene glycol (PEG) and lactulose increased defecation frequency and decreased faecal incontinence frequency. However, in 30–50% of the patients using these compounds, adverse side-effects were observed such as: abdominal pain, bloating, flatulence, diarrhoea, nausea and bad taste [4,5]. Low compliance is probably of major importance with respect to the low percentage of children cured after 6 months of laxative treatment, suggesting that development of new treatment strategies is of great importance.

### Rationale for the efficacy of the fermented dairy product containing Bifidobacterium lactis *DN-173 010 *in childhood constipation

Probiotics are defined as live micro-organisms which when administered in adequate amounts confer a health benefit on the host [6]. It is suggested that there is a dysbiosis in the gut flora in constipated patients which might be improved by probiotics [7]. This is important because colonic microflora influences the peristalsis of the colon. Furthermore, Bifidobacteria can lower the pH of the colon by producing lactic, acetic and other acids. A lower pH enhances peristalsis of the colon and subsequently decreases colonic transit time which is beneficial in the treatment of constipation [7,8]. Activia^® ^is a fermented milk manufactured with lactic cultures including yoghurt starter cultures (*Lactobacillus bulgaricus *and *Streptococcus thermophilus*) and a specific probiotic strain, *Bifidobacterium lactis *DN-173 010. Recently, studies have shown that this fermented milk significantly decreased transit time in young and elderly healthy adults [9-13] and in constipation-predominant IBS patients [14]. Moreover, a recent randomised double-blind controlled trial in IBS patients with constipation showed a significant increase, as compared to controls, in stool frequency over the 6-weeks product consumption in the subgroup of patients with a defecation frequency <3/week [15]. Another recent randomised double-blind controlled study demonstrated a significant increase of stool frequency in constipated women with a defecation frequency <3/week following consumption of the fermented milk for 2 weeks compared to control group [16]. In a small pilot study in The Netherlands, 8 consecutive children with untreated constipation with a defecation frequency < 3 per week and hard stools were treated with the fermented milk 2 times/day for one month. A normalization of the defecation frequency and improvement from hard to soft stools were seen in five of them. In the three other children no improvement was found. None of the children reported side effects. Based on these data, a multi-centre RCT is required to assess whether Activia^® ^is effective in the treatment of childhood constipation. We hypothesize that the compliance increases by taking fermented milk containing *Bifidobacterium lactis *DN-173 010. If this study indeed shows a significant better effect of active product compared to control product, this will change the treatment of newly diagnosed children with constipation. This paper describes the rationale and the design of this study.

## Methods/design

### Study objectives

The study objective is to show that the fermented milk containing *Bifidobacterium lactis *DN-173 010 is effective in increasing defecation frequency after 3 weeks of product consumption in children with functional constipation.

### Primary endpoint

The primary endpoint is the stool frequency change from baseline to 3 weeks of product consumption.

### Secondary endpoints

Secondary endpoints are:

- Stool frequency over 3 weeks and at week 1 and 2 of product consumption.

- Stool consistency over 3 weeks and per week of product consumption.

- Frequency of episodes of faecal incontinence over 3 weeks and per week of product consumption.

- Pain during defecation over 3 weeks and per week of product consumption.

- Digestive symptoms: abdominal pain and flatulence over 3 weeks and per week of product consumption.

- Adverse effects: nausea, diarrhoea and bad taste over 3 weeks and per week of product consumption.

- Intake of Bisacodyl over 3 weeks and per week of product consumption.

- Rate of success with success defined as 3 or more bowel movements per week and less than 1 faecal incontinence episode in 2 weeks over the last 2 weeks of product consumption

- Rate of responders with a responder defined as a subject who reports a stool frequency = 3 on the last week of product consumption

### Design

Double-blind, placebo-controlled randomised multicentre, two nation (the Netherlands and Poland) trial.

### Setting

Children able to participate in this study will be recruited in two countries in Europe: from three academic hospitals (Academic Medical Centre Amsterdam, University Hospital Groningen, The Netherlands and the Medical University of Warsaw, Poland), 12 Dutch non-academic hospitals and general practitioners (region of Rotterdam) in The Netherlands.

### Patients

A total of 160 functional constipated children, boys and girls, aged from 3 to 16 years will be randomised.

### Study products

Test product: fermented milk Activia^® ^(125-g pot) manufactured with lactic cultures including yoghurt starter cultures (*Lactobacillus delbrueckii *ssp. *bulgaricus *and *Streptococcus thermophilus*) [at least 1,2 × 10^9 ^colony forming units (cfu) per pot] and a specific strain, *Bifidobacterium lactis *DN-173 010 [at least 6 × 10^9^cfu per pot].

Control: milk-based non-fermented dairy product (125-g pot) without probiotics and with low content of lactose < 4 g/pot as in the test product.

Every patient has to take two pots of study products per day (test or control) to be consumed in two times (one at the breakfast and one at the evening meal) for 3 consecutive weeks. During the entire study, all children are instructed to try to defecate on the toilet for 5–10 minutes after each meal (3 times a day) and to complete daily a standardized bowel diary. They were also instructed not to consume other fermented dairy products or yoghurts during all the study.

### Eligibility criteria

#### Inclusion criteria

-Children (boys and girls) aged from 3 to 16 years

-Children with a diagnosis of functional constipation according to Rome III criteria [17]:

- subjects must present defecation frequency < 3/week and:

- subjects must present 1 or more of the following criteria:

faecal incontinence > 1/week

large amount of stools which clog the toilet

painful defecation

withholding behaviour

abdominal or rectal faecal impaction upon physical examination

-Children with a diagnosis of functional constipation according to Rome III criteria fulfilled for the last 2 months

-Children with usual consumption of dairy products and ready to consume 2 pots per day

-Children having given written consent to take part in the study (in The Netherlands: children and parents for children above 12 years and only parents for children under 12 years; in Poland: children and parents for children above 16 years and only parents for children under 16 years).

#### Non-inclusion criteria

-Children with a diagnosis of IBS according to Rome III criteria

-Children treated for constipation less than 2 weeks before intake in the study

-Children with mental retardation or metabolic disease (hypothyroidism)

-Children with Hirschsprung's disease or spinal anomalies or anorectal pathology

-Children who underwent gastro-intestinal surgery

-Children with functional non-retentive faecal incontinence

-Children with lactose intolerance or known allergy to product component (milk protein for example)

-Children who started a medication with antibiotics in the prior month

-Children receiving medication influencing gastrointestinal motility (for examples Cisapride, Motilium, Erythromycin, laxatives, Loperamide)

During the study it is not allowed to consume any other fermented dairy product or yoghurt.

#### Randomisation criteria (checked at visit 2)

After inclusion in the study, all patients with a defecation frequency < 3 per week between visit 1 and visit 2 and who did not use any laxative between V1 and V2 are randomised at visit 2.

### Ethics, informed consent

This study is conducted in accordance with the principles of the Declaration of Helsinki and 'good clinical practice' guidelines (ICH E6). The independent ethics committee of all participating hospitals approved the final protocol. Written informed consent is obtained from the patient and/or parents before inclusion in the trial at visit 1.

### Safety

To date, no adverse events associated with the consumption of the fermented milk containing *Bifidobacterium lactis *DN-173 010 were observed in clinical studies done with this product. During consumption of the study product, both the patient and medical staff are asked to register any potential side effect or adverse event. All adverse events will be monitored and discussed by an independent Clinical Research Organisation.

### Statistical analysis

#### Intention-to-treat

The analysis will be performed on the basis of an intention-to-treat (ITT) population and with respect to ITT principles. Also a per-protocol (PP) analysis will be performed.

#### Sample size

We assume that the success percentage in the intervention group (fermented milk containing *Bifidobacterium lactis *DN-173 010, toilet training, bowel diary) will be 35% and the success percentage in the control group (acidified milk without ferments, toilet training, bowel diary) will be 15%, and "alpha" is 0.05 and the power ("beta") is 80%. The choice of 15% is justified by a previous study of van der Plas et al. showing that 15% of children with untreated chronic defecation problems can be helped by an approach of toilet training and a daily bowel diary alone[18]. The total number of randomised subjects in this study has to be 146. To allow withdrawal a number of 160 subjects will be randomised. This means each group consists of 80 subjects. The number of randomised subjects per country will be 80 (± 20%).

### Randomisation

The assignment of subjects to test or control groups will be carried-out by randomisation in well balanced blocks performed by Danone Research prior to the study onset. These randomisation lists (one per country) used for assigning each subject to a treatment group will be prepared and kept confidentially. It will be forwarded to the person responsible of the preparation of study products and their labelling. Subjects will be then included sequentially in both countries per randomisation lists (incrementally by randomisation number by the IWRS system).

### Blinding

The two treatments, fermented milk containing *Bifidobacterium lactis *DN-173 010 and control product, are identical in weight, colour, smell, taste and package. All doctors, research staff and patients involved are unaware of the treatment administered to the patient.

### Treatment program

**Figure 1:**
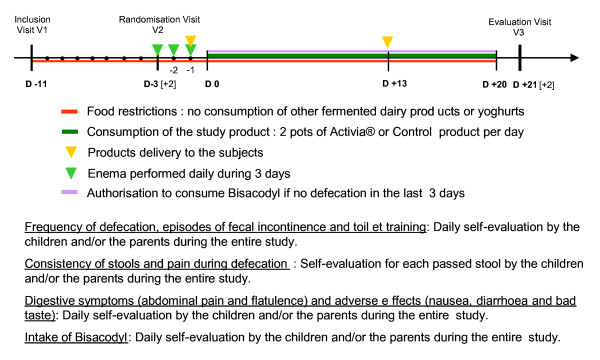
**Study planning in days (D-11 to D+21)**.

See figure 1 for the study planning. The total duration of the study is approximately 5 weeks for each subject. Each patient will attend 3 clinic appointments: Inclusion visit V1 (baseline), randomisation visit (V2) and clinical evaluation at weeks 3 (V3). When a child is eligible for inclusion, a list of non-authorized products will be discussed with the child and parents at visit 1. The first 8 days will be used to obtain baseline values for the outcome parameters. After 3 days of enemas, the 3 subsequent weeks (day 0 to day 20) will constitute the treatment phase. The subjects will receive and consume 2 products (test or control product) per day. Between days 7 and 10, the subjects will receive new products, to be consumed during the second part of the treatment period, to ensure consumption before end of shelf-life of the products. They will cease consumption of the study product at the end of week 3 (day 20) and will then have their last visit on day 21. During the treatment phase, patients can take Bisacodyl 5 mg if they have no defecation for 3 consecutive days.

Frequency of defecation, frequency of faecal incontinence episodes, frequency of toilet training, self-evaluation of digestive symptoms (abdominal pain and flatulence) and of adverse effects (nausea, diarrhoea and bad taste) will be assessed daily during the entire study using a subject diary. Stool consistency and pain during defecation will be assessed for each passed stool using the same paper diary. The subject's diary will be also used to record the daily consumption of the study products, any unauthorised products and Bisacodyl and any other concomitant treatment.

### Monitoring

Independent Clinical Research Associates (CRA) will visit at least twice every site in order to monitor all the patients' data.

### Follow-up

After finishing the study, all children will get an appointment at the outpatient clinic in their hospital for treatment of their constipation.

## Discussion

Only children with a defecation frequency < 3/week will be included in this study. This seems inconsistent with the ROME-III definition for functional constipation, since 2 out of 6 defecation related criteria should be present. However, in our experience approximately 100% of children with a defecation frequency < 3/week fulfil one or more other symptoms of the Rome definition for functional constipation. An advantage of this strategy is that a more homogenous population of children with functional constipation will be included. Furthermore, a recent double-blind controlled trial showed beneficial effect of the fermented milk containing *Bifidobacterium lactis *DN-173 010 in increasing stool frequency in IBS patients with constipation and a defecation frequency <3/week and another one showed an increase in stool frequency in constipated women also with a defecation frequency <3/week following consumption of this product.

Recently 4 human double-blind randomised studies using 1–3 portions of fermented milk containing *Bifidobacterium lactis *strain DN-173 010 all reported a significant reduction of transit time in healthy subjects, especially in those with slow transit[7,9,11-13], and also in IBS patients with constipation[14]. No adverse effects associated with the consumption of the fermented milk containing *Bifidobacterium lactis *DN-173 010 were observed in clinical studies done with this product. In the healthy adults, three portions per day proved to be more effective than two, whereas two portions appeared more effective than one. In this study we have chosen for two portions of fermented milk containing *Bifidobacterium lactis *DN-173. In these studies a reduction in transit time was observed within two weeks. Therefore we expect that three weeks of ingestion of the fermented milk containing *Bifidobacterium lactis *DN-173 010 are sufficient to determine its effectiveness.

Bisacodyl is a laxative of the triarylmethane group, which is hydrolyzed in the bowel by local enzymes into the active agent bis-(*p*-hydroxyphenyl)-pyridyl-2-methane (BHPM). Bisacodyl, in contrast to polyethylene glycol and lactulose, directly stimulates colonic peristalsis and does not interfere with the microflora of the gut. Therefore Bisacodyl has been chosen as rescue medication in case children do not defecate for three consecutive days during the study period.

## Conclusion

This is the first double-blind, placebo-controlled randomised multicentre trial that aims to show that the fermented milk containing *Bifidobacterium lactis *DN-173 010 is effective in increasing stool frequency after 3 weeks of product consumption in children with functional constipation.

## Abbreviations

BHPM: bis-(*p*-hydroxyphenyl)-pyridyl-2-methane; CFU: colony-forming unit; CRA: Clinical Research Associates; IBS: irritable bowel syndrome; ICH: International Conference on Harmonisation; ITT: intention-to-treat; IWRS: Interactive Web Response System; PEG: polyethylene glycol; PP: per protocol; RCT: randomised controlled trial.

## Competing interests

The authors declare that they have no competing interests.

## Authors' contributions

MMT drafted the manuscript. MAB, AA and HS edited the manuscript. All authors participated in the design of the study. All authors read and approved the final manuscript.

## Pre-publication history

The pre-publication history for this paper can be accessed here:


